# Super-taxon in human microbiome are identified to be associated with colorectal cancer

**DOI:** 10.1186/s12859-022-04786-9

**Published:** 2022-06-21

**Authors:** Wei Dai, Cai Li, Ting Li, Jianchang Hu, Heping Zhang

**Affiliations:** 1 Department of Biostatistics, Yale University School of Public Health, 300 George Street, Ste 523, New Haven, CT 06511 USA; 2 Department of Biostatistics, St. Jude Children’s Research Hospital, Memphis, TN 38105 USA; 3 Department of Applied Mathematics, The Hong Kong Polytechnic University, Hong Kong, China; 4 Institut für Medizinische Biometrie und Statistik, Universität zu Lübeck, Universitätsklinikum Schleswig-Holstein, Campus Lübeck, Lübeck, Germany

**Keywords:** Colorectal cancer, Microbiota-disease association studies, Microbiome joint effects, Super-Taxon

## Abstract

**Background:**

Microbial communities in the human body, also known as human microbiota, impact human health, such as colorectal cancer (CRC). However, the different roles that microbial communities play in healthy and disease hosts remain largely unknown. The microbial communities are typically recorded through the taxa counts of operational taxonomic units (OTUs). The sparsity and high correlations among OTUs pose major challenges for understanding the microbiota-disease relation. Furthermore, the taxa data are structured in the sense that OTUs are related evolutionarily by a hierarchical structure.

**Results:**

In this study, we borrow the idea of super-variant from statistical genetics, and propose a new concept called super-taxon to exploit hierarchical structure of taxa for microbiome studies, which is essentially a combination of taxonomic units. Specifically, we model a genus which consists of a set of OTUs at low hierarchy and is designed to reflect both marginal and joint effects of OTUs associated with the risk of CRC to address these issues. We first demonstrate the power of super-taxon in detecting highly correlated OTUs. Then, we identify CRC-associated OTUs in two publicly available datasets via a discovery-validation procedure. Specifically, four species of two genera are found to be associated with CRC: Parvimonas micra, Parvimonas sp., Peptostreptococcus stomatis, and Peptostreptococcus anaerobius. More importantly, for the first time, we report the joint effect of Parvimonas micra and Parvimonas sp. (p = 0.0084) as well as that of Peptostrepto-coccus stomatis and Peptostreptococcus anaerobius (p = 8.21e-06) on CRC. The proposed approach provides a novel and useful tool for identifying disease-related microbes by taking the hierarchical structure of taxa into account and further sheds new lights on their potential joint effects as a community in disease development.

**Conclusions:**

Our work shows that proposed approaches are effective to study the microbiota-disease relation taking into account for the sparsity, hierarchical and correlated structure among microbes.

**Supplementary Information:**

The online version contains supplementary material available at 10.1186/s12859-022-04786-9.

## Background

The human microbiome is an integral component in the maintenance of immune system and health [1]. It has been shown to be associated with many diseases including diabetes, obesity and colorectal cancer [2–4].

In microbiome studies, 16S ribosomal RNA (rRNA) sequencing approach, which profiles bacterial community by sequencing the 16S rRNA marker gene, has been widely used. Microbial count data are represented using operational taxonomic units (OTUs) from 16S rRNA sequencing. The study of microbiome with OTUs has led to successful findings for many complex diseases. For instance, Duvallet et al. [5] conducted meta-analysis over 28 public case–control gut microbiome studies spanning ten diseases to identify disease-specific and shared microbiotas. Nolan-Kenney et al. [6] studied association between smoking and gut microbiome in Bangladesh. However, the effect attributed to microbiome factors is still far from well understood [7]. One important feature of the count data is that not all taxa may be present in each sample which leads to lots of zero values for some of the OTUs [8]. The sparsity of taxa counts certainly bring challenges in the association study of microbiome factors. The other impediment is the focus on the joint effect of individual OTUs. Although there has been growing interests to study the community of bacteria, viruses, and microbial eukaryotes as a whole and to identify microbe–microbe interactions for complex diseases [7, 9], surprisingly, less progress has been made in this direction due to the high correlations among individual OTUs. On top of that, the OTUs are related to one another by an evolutionally hierarchical structure. Appropriate use of this hierarchical information is expected to offer more insights.

There are previous studies on finding CRC associated microbes. Most studies apply a univariate test to detect significant differences in relative abundances of OTUs individually, such as t-test or nonparametric Wilcoxon test [10, 11]. These tools are unable to account for the sparsity issue in OTUs data. Zero-beta inflated regression is another popular method for univariate association between individual OTU and disease phenotype [12]. However, all the univariate tests ignore the correlations among OTUs, which might lead to an inefficient detection. Another category of finding CRC-associated microbes focuses on predictive modeling, such as random forest [4, 5], which regards an OTU as CRC-associated if it is kept in the final predictive model after tree pruning. However, these analyses focus more on prediction accuracy instead of associations which is a potential gap we aim to fill in this work. For finding the effects for a community or set of OTUs on diseases, unweighted UniFrac and weighted UniFrac distance metrics analysis [13] are normally carried out, however, these methods fail to quantify the contribution of each individual OTU.

To overcome the issues mentioned above, we borrow the idea of super-variant which has been adopted in genome-wide association studies and brain imaging genetics [14–16]. In this study, we propose a new concept called super-taxon for microbiota-disease association studies. Super-taxon, a combination of taxonomic units, is constructed based on hierarchical information of taxa at different levels according to various scientific relevance. For example, a super-taxon can be a genus that consists of OTUs at species-level. The super-taxon is expected to reflect both the marginal and joint effects of contributing OTUs on diseases. It has been shown that the super-variants in genetic studies are easy to detect, and the aggregated signals are stable in their associations with the responses [14–16]. By exploiting the proposed methods, not only can we find the association for a microbiome community, but also report the top contributing individual OTUs.

We apply our method to two datasets for colorectal cancer, Baxter et al. [4] and Zeller et al. [10] respectively. Our methods identify and validate two significant genera. More importantly, our methods can use the hierarchical information to go beyond genera-level for identifying the joint effects of OTUs at species-level: two sets of OTUs that might jointly influence CRC, one consisting of Peptostreptococcus stomatis and Peptostreptococcus anaerobius (p = 8.21e-06), the other one including Parvimonas micra and Parvimonas sp. (p = 0.0084), which sheds light on the future research to study the joint effects of microbial community on diseases.

## Materials and methods

### Methodology details

**Fig. 1:**
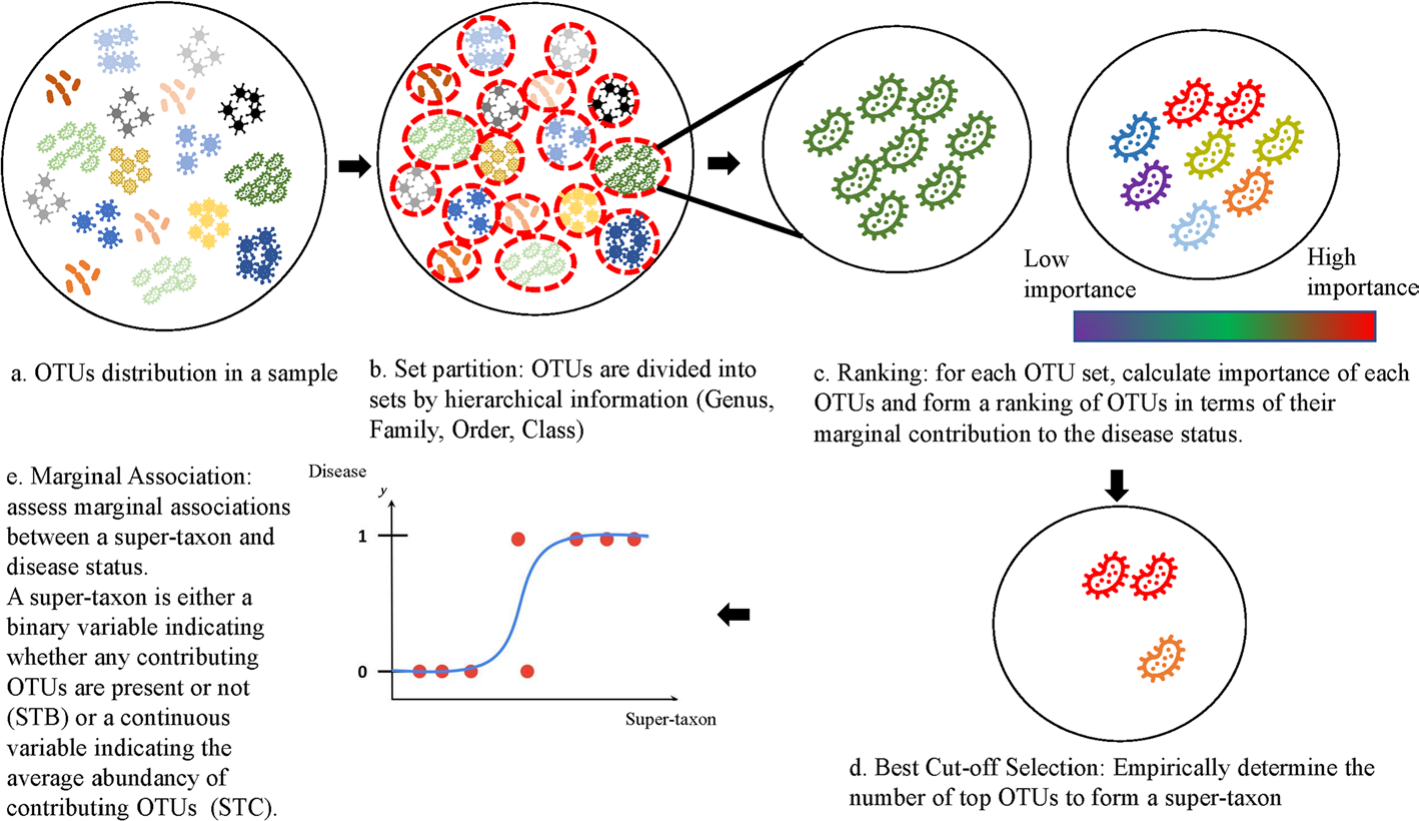
Method Overview. **a** OTUs in a sample are displayed. **b** OTUs are divided into sets/blocks by biological group (Genus, Family, Order, Class). **c** Within each set, a tree-based method is utilized to obtain the importance measure of each OTU and form a ranking of OTUs in terms of their marginal contribution to the disease status. **d** Empirically determine the number of top OTUs to form a super-taxon. **e** Top OTUs within each set/block are then aggregated into a super-taxon (STB and STC are both considered for OTU presence or abundance)

The method consists of four steps, and a flowchart of the method is presented Fig. 1. In the first step (Fig. 1b), OTUs are divided into OTU sets or blocks which will be discussed in detail later. In the second step (Fig. 1c), within each block, a tree-based approach will be used to generate a ranking of OTUs based on the depth importance measures that account for their marginal contribution to the phenotype. In the third step (Fig. 1d), we empirically determine the number of top OTUs to form a super-taxon as describe in detail as follows. In the last step (Fig. 1e), top OTUs within each local block are then aggregated into a super-taxon and marginal logistic regression is applied to associate the super-taxon with the phenotype. We describe details in each step as follows.

#### Set partition

OTUs are first divided into sets by their sequence similarity or biological clustering through RDP classifier [17]. The hierarchical information will be utilized to assist in partition at different levels (Genus, Family, Order, Class).

#### Ranking

We consider the following generalization of the logistic model,

$$\log \frac{{P\left( {Y_{i} = 1} \right)}}{{P\left( {Y_{i} = 0} \right)}} = F\left( {v_{i} , z_{i} } \right),$$

where $F$ is a function not limited to be linear, $i$ is subject index ($i = 1, \ldots ,n$), $Y_{i}$ indicates the disease status, $v_{i} = \left( {v_{i,1g} , \ldots ,v_{i,Jg} } \right)^{T}$ includes $J_{g}$ OTU features in an OTU set $g$ for. $i$-th subject, and $z_{i}$ denotes all confounding covariates to be adjusted. To estimate the unknown function $F$, we consider the tree-based method which allows potential nonlinear relations and any possible interactions between OTUs.

We adopt the ranking approach and modify the aggregation approach in Hu et al. [14] to identify the super-taxon of OTUs. It is based on the ranked but generally weak association between an individual OTU and a disease of interest. Within each partitioned set, OTUs are then ranked by their importance in terms of disease discrimination ability. To account for joint effects of multiple OTUs, the importance of each OTU is measured by the so-called depth-importance in a random-forest framework [18, 19], which is a proxy to its effect size and leads to an ordering of OTUs within the set. Specifically, for a set of OTU features $g$, we construct forest $f$ consisting of a total number of $\left| f \right|$ trees. Each tree in the forest is built without pruning based on a randomly selected subset of variants in $g$. We define $v_{jg} = \left( {v_{1,jg} , \ldots ,v_{n,jg} } \right)^{T}$ as the $j$-th OTU in $g$-th set across subjects. Then, depth importance score of $v_{jg}$ in tree $T$ is defined as

$$V_{T} \left( {v_{jg} } \right) = \sum\limits_{{t \in T,{ }t{ }is{ }split{ }by{ }v_{jg} }} {2^{{ - L_{t} }} G_{t} } ,$$

where $L_{t}$ is the depth of node $t$ and $G_{t}$ is the χ.^2^ independence test statistics of node $t$. The calculation of depth importance considers both effect size and depth of an OTU in the tree. We refer the readers to previous random-forest framework for more detailed calculations on depth importance score [18, 19]. Then the overall depth importance score for $v_{jg}$ is given by

$$V_{f} \left( {v_{jg} } \right) = \frac{1}{\left| f \right|}\sum\limits_{T \in f} {V_{T} \left( {v_{jg} } \right)} ,$$

overall $\left| f \right|$ trees in the forest $f$. With the overall depth importance score $V_{f} \left( {v_{jg} } \right)$ calculated, we can obtain the ordering of OTUs within the set $g$. Let $d_{jg}$ be the index of the OTU with the $j$-th largest depth importance score for $g$-th set. For example, if we have five OTUs in set $g$, i.e., $J_{g} = 5$ and overall depth importance scores for those OTUs are $V_{f} \left( {v_{g} } \right) = \left( {V_{f} \left( {v_{1g} } \right),V_{f} \left( {v_{2g} } \right),V_{f} \left( {v_{3g} } \right),V_{f} \left( {v_{4g} } \right),V_{f} \left( {v_{5g} } \right)} \right)^{T} = \left( {0.2, 0.4,0.3,0.6,0.1} \right)^{T}$, the OTU with the largest overall depth importance score is the 4-th OTU, i.e., $d_{1g} = 4$. Thus, we end up with ordered OTUs and their ranks $d_{g} = \left( {d_{1g} , \ldots ,d_{5g} } \right)^{T} = \left( {4,2,3,1,5} \right)^{T}$. For $i$-th subject, let $v_{i,djg}$ be the value of $d_{jg}$-th OTU in $g$-th set. Define

$$x_{ig} = \left\{ {\begin{array}{*{20}c} {\mathop {\min }\limits_{{1 \le j \le J_{g} }} \left\{ {j: v_{{i,d_{jg} g}} > 0} \right\}, if \exists v_{{i,d_{jg} g}} > 0,} \\ {J_{g} + 1, otherwise,} \\ \end{array} } \right.$$

The creation of $x_{ig}$ aims to provide guidance on selecting best cut-off in the following steps.

#### Best cut-off selection

To select the top OTUs, we first need to define how to form a super-taxon from top OTUs. There are two ways of formation. One is called super-taxon for binary features (STB), where the OTUs data are turned into binary one, indicating that we only care about the presence or absence of an OTU. The other is named as super-taxon for continuous features (STC), where the OTUs data are kept as original and abundance information can be utilized. Both STB and STC are proposed to deal with the sparsity issue and complementary each other under various sparsity levels. For STB, the variable is turned into binary for each threshold; that is, for a threshold $c$,

$$S_{ig} = I\left( {x_{ig} < c} \right)$$,

where $I\left( \cdot \right)$ is the indicator function, and $c \in \left\{ {x_{1g} , \ldots , x_{ng} } \right\}$. For STC, the variable is turned into mean of non-zero features for each threshold; that is, for a threshold $c$,

$$S_{ig} = {{\sum\limits_{{d_{jg} \le c}} {v_{igdjg} } } \mathord{\left/ {\vphantom {{\sum\limits_{{d_{jg} \le c}} {v_{igdjg} } } {\sum\limits_{{d_{jg} \le c}} {I\left( {v_{igdjg} > 0} \right)} }}} \right. \kern-\nulldelimiterspace} {\sum\limits_{{d_{jg} \le c}} {I\left( {v_{igdjg} > 0} \right)} }},$$

where $I\left( \cdot \right)$ is the indicator function, and $c \in \left\{ {x_{1g} , \ldots , x_{ng} } \right\}$. Both transformations are designed to reduce the effect from the unobserved taxa within the set. While STB focuses more on the effect of taxa’s presence, STC further takes the expression level into account. For different sparsity levels of taxa, STB and STC are expected have their own advantages. With the formed super-taxon, a univariate logistic regression is carried out to investigate its effect, and the final threshold is the one gives the smallest p-value among all possible thresholds.

#### Marginal associations

With selected OTUs, a super-taxon can be constructed with those OTUs in the depth-importance-ordered OTU list and the total number of OTUs used to form the super-taxon is the same as the final threshold. The logistic regression is performed to assess the association and effect between a super-taxon and disease status. We refer readers to Hu et al. [14] for more details.

### Simulation setup

In the simulation, performance of our proposed method in detecting differentially expressed OTU features was evaluated. We adopt the same model for generating the synthetic OTUs data as in Osborne et al. [20] to account for high correlations among OTUs. We compare our method with four classical methods listed below.

1. DESeq2 [21]: an RNA-seq based method that models the observed OTU abundances using negative binomial (NB) distribution.
2. Zero-inflated beta regression (ZIBR): an extension of the generalized linear model (GLM) approach that takes sparse nature of OTU data into account [12].
3. Analysis of compositions of microbiomes with bias correction (ANCOM-BC) method [22]: it models observed abundances using an offset-based log-linear model. It was shown to control the false discovery rate (FDR) and competed very well with other methods in terms of power in a review paper on comparing statistical methods in differential abundance analysis for microbiome data [22].
4. The original method, super-variants, proposed in Hu et al. [14]: it captures the potential interactions among OTUs through random-forest model for binary features without splitting OTUs into sets, which can be considered as a special case of our approach based on OTU level.

Specifically, to mimic the dependence among OTUs, we simulate correlated OTUs from the following model [20].

$$n_{i} \sim N\left( {3000, 250} \right)$$

$$X_{i} \sim {\text{Multinomial}}\left( {h_{i} ,n_{i} } \right)$$

$$h_{i} \sim Dirichlet\left( {\alpha_{i} } \right)$$

$$\log \left( {\alpha_{i} } \right) \sim MVN\left( {Y_{i} B + B_{0} ,\Omega } \right)$$

where $Y$ is a binary covariate to mimic the case-control study, with $Y = 0$ for $\frac{N}{2}$ subjects and $Y = 1$ for the other $\frac{N}{2}$ subjects. Of them, $n_{i}$ is the total count in $i$-th sample. $h_{i}$ is the relative abundances and $\alpha_{i}$ is the absolute abundance. Through ${\Omega }$, we can capture the correlations among OTUs. Following Osborne et al. [20], we set $B_{0j} \sim U\left( {6,8} \right)$ with probability of 0.2 and $B_{0j} \sim U\left( {2,4} \right)$ with probability of 0.8, which allows that some variables have larger counts and others to be sparser, as common in microbiome data. This controls the average sparsity for OTUs to be around 0.6 in the simulated dataset.

For evaluating type I error rate, we set $B$ to 0. ${\Omega }$ is generated from the random graph as described in Osborne et al. [20]. The simulation is carried with $N = 800$ subjects and 1000 OTUs into 20 sets or blocks (50 OTUs per block). Since there are multiple blocks, we evaluate type I error rate through family-wise error rate (FWER), where an error is made if any set is rejected. To control the family-wise error rate, we divide the data into two even sets. The first set is used for performing steps a–d in Fig. 1 while the second set is applied to the final step in Fig. 1. For the competing four methods, since they are only able to perform univariate testing for each OTU, the FWER in this case is calculated if a set is rejected if any OTU in the set is rejected. The FWER is calculated at nominal level 0.05.

In terms of power evaluation, as in the previous setting, we simulate 800 samples with 1000 OTUs into 20 blocks (50 OTUs per block). Of those, only 40 OTUs are associated with the binary covariates and scattered evenly in the first 4 blocks (10 true OTUs / block). We simulate elements in $B$ as follows:

1. For 40 binary covariates associated OTUs, $B_{j} \sim U\left( {0.5,1} \right)$ with probability of 0.5 and $B_{j} \sim U\left( {0.1,5} \right)$ with probability of 0.5.
2. For the rest of OTUs, $B_{j} = 0$.

$\Omega$ is generated from the random, hub and cluster graph as described in Osborne et al. [20]. The power performances are evaluated based on two perspectives. First, we evaluate the block-level identification rate, defined as the number of times a block is selected over 500 repeats. For ZIBR, ANCOM-BC and super-variants, since they all generate results for single OTU-level testing, a block is regarded as selected if any OTU in the block is selected. We also evaluate the sensitivity (TP/(TP + FN)), specificity (TN/(FP + TN)), precision (TP/(TP + FP)) in terms of block-level selection. Second, for assessing the OTU-level identification, we also report the average sensitivity, specificity and precision over repeats.

To further investigate the effects of sparsity levels on methods performance, more numerical studies are conducted to compare the performances of STB and STC under various sparsity levels. The goal of these studies is to provide some empirical experience about the scenarios where one method gains over the other. Specifically, by varying sparsity levels, we investigate on the cases where STB outperforms STC and vice versa to provide some guidance for practical usage.

The same model is used to simulate correlated OTUs. Again, we simulate 800 samples with 1000 OTUs into 20 blocks (50 OTUs per block). Of those, only 40 OTUs are associated with the binary covariates and scattered evenly in the first 4 blocks (10 true OTUs / block). ${\Omega }$ is generated from random graph. We simulate elements in $B$ as follows:

1. For 10 binary covariates associated OTUs in 1st block, $B_{j} \sim U\left( {0.1,0.5} \right)$.
2. For 10 binary covariates associated OTUs in 2nd block, $B_{j} \sim U\left( { - 0.5, - 0.1} \right)$
3. For 10 binary covariates associated OTUs in 3rd block, $B_{j} \sim U\left( {0.5,1} \right)$
4. For 10 binary covariates associated OTUs in 4th block, $B_{j} \sim U\left( { - 1, - 0.5} \right)$
5. For the rest of OTUs, $B_{j} = 0$.

Additionally, we set $B_{0j} \sim U\left( {a,a + 2} \right)$ with probability of 0.2 and $B_{0j} \sim U\left( {2,4} \right)$ with probability of 0.8, where $a$ controls sparsity level. The sparsity varies from 0.4 to 0.8 with $a$ ranging from 4 to 8. The performance is evaluated through block-level identification rate, sensitivity, specificity and precision in terms of block-level selection.

## Simulation results

### Type-I error and power results

For Type I error evaluations, the FWERs for STB and STC are 0.0378 and 0.0622 respectively, indicating that both methods can control the FWER around 0.05. For the other three competing methods, ZIBR has FWER of 0.0752, which has slightly inflation. The super-variant approach from Hu et al. [14] controls FWER well with value of 0.0449. ANCOM-BC has FWER of 0 while DESeq2 has FWER of 0.6894, indicating that DESeq2 fails to control Type I error and thus, will be excluded from later power comparison. Since both STB and STC as well as the other three competing methods, ZIBR, ANCOM-BC and super-variants control the FWER at a desired level,

With regards to power evaluations, the block-level results are shown in Tables 1 and 2. Our methods outperform the original super-variant approach in all scenarios. For comparison with other approaches, while there is no all-time winner, our proposed methods perform better than ZIBR in terms of the identification rates of block level in most of the scenarios. More importantly, the averaged identification rates of the proposed methods are comparable to or higher than those of ZIBR. This pattern is particularly profound for the scenarios of hub and cluster. In addition, across three graph structures, both STB and STC can achieve higher sensitivity than ZIBR while the specificity and precision remain comparable. Although ANCOM-BC achieves high identification rates, but it turns out to have extremely low specificity and precision of block level in all scenarios which implies that it may predict everything into positives without providing useful information.

**Table 1:** Block-level identification rate over 500 replications for four true blocks under three graph structures

Method	Graph type	Block 1	Block 2	Block 3	Block 4	Average (SD)
STB	Random	0.8289	0.9918	1	0.8598	0.9201 (0.0885)
STC		0.9401	1	0.6736	1	0.9034 (0.1558)
ZIBR		0.8223	0.8595	0.9814	0.9835	0.9117 (0.0831)
ANCOM-BC		1	1	1	1	1 (0)
Hu et al. [14]		0.0020	0.4633	0.1388	0	0.1510 (0.2181)
STB	Hub	0.7478	0.7917	1	0.9364	0.8690 (0.1188)
STC		0.9670	1	1	1	0.9917 (0.0165)
ZIBR		0.6865	0.7726	1	1	0.8648 (0.1601)
ANCOM-BC		1	1	1	1	1 (0)
Hu et al. (2020)		0.0082	0.2238	0.1458	0	0.0944 (0.1091)
STB	Cluster	0.7868	1	1	1	0.9467 (0.1066)
STC		0.9510	1	0.9787	0.7975	0.9318 (0.0918)
ZIBR		0.7361	0.7961	0.7682	0.9506	0.8128 (0.0951)
ANCOM-BC		1	1	1	1	1 (0)
Hu et al. [14]		0.0040	0.3300	0.1360	0	0.1175 (0.1551)

$\Omega$ is set to reflect the correlations among OTUs. Random graph structure indicates that OTUs are correlated with each other randomly. The hub and cluster graphs capture some aspects of biological networks, such as highly connected nodes and community structure. More details can be found in Osborne et al. [20]

**Table 2:** Average of sensitivity, specificity, precision and standard deviations for block-level identification over 500 replications

Method	Graph type	Sensitivity (SD)	Specificity (SD)	Precision (SD)
STB	Random	0.9201 (0.1178)	0.9996 (0.0049)	0.9988 (0.0157)
STC		0.9034 (0.1219)	1 (0)	1 (0)
ZIBR		0.9117 (0.1228)	1 (0)	1 (0)
ANCOM-BC		1 (0)	0 (0)	0.2 (0)
Hu et al. [14]		0.1510 (0.1255)	0.9719 (0.0326)	0.5806 (0.4815)
STB	Hub	0.8690 (0.1250)	1 (0)	1 (0)
STC		0.9917 (0.0447)	0.9869 (0.0255)	0.9581 (0.0814)
ZIBR		0.8648 (0.1404)	1 (0)	1 (0)
ANCOM-BC		1 (0)	0 (0)	0.2 (0)
Hu et al. [14]		0.0945 (0.1213)	0.9588 (0.0307)	0.3634 (0.4739)
STB	Cluster	0.9467 (0.1025)	0.9975 (0.0123)	0.9919 (0.0395)
STC		0.9318 (0.1115)	1 (0)	1 (0)
ZIBR		0.8128 (0.1340)	1 (0)	1 (0)
ANCOM-BC		1 (0)	0 (0)	0.2 (0)
Hu et al. [14]		0.1175 (0.1259)	0.9655 (0.0323)	0.4630 (0.4966)

Different graph types are set to reflect various correlation structures among OTUs. Random graph structure indicates that OTUs are correlated with each other randomly. The hub and cluster graphs capture some aspects of biological networks, such as highly connected nodes and community structure. More details can be found in Osborne et al. [20]

In terms of the OTU-level identification (Table 3), starting from sensitivity, STB/STC and ZIBR are comparable, while ANCOM-BC has higher values. For specificity, STB/STC and ZIBR achieve high values while specificity for ANCOM-BC is low. Regarding precision, STB and STC can achieve high precision while ZIBR and ANCOM-BC do not perform equally well in this case. This may be because both ZIBR and ANCOM-BC do not account for the correlations among OTUs, which might be a disadvantage compared with our proposed method. Similar to block-level results, ANCOM-BC can achieve high sensitivities but has lower specificity and precision, which impedes its usefulness. Across all scenarios, STB and STC show significantly better performance than the original super-variant approach, which indicates that the utilization of hierarchical information in our methods helps with OTUs discovery.

**Table 3:** Average of sensitivity, specificity, precision and standard deviations for OTU-level identification over 500 replications

Method	Graph type	Sensitivity (SD)	Specificity (SD)	Precision (SD)
STB	Random	0.2815 (0.0609)	0.9979 (0.0015)	0.9073 (0.0438)
STC		0.1377 (0.0724)	0.9993 (0.0011)	0.9528 (0.0637)
ZIBR		0.1463 (0.0486)	1 (0)	0.5083 (0.2771)
ANCOM-BC		0.7225 (0.0152)	0.6084 (0.0078)	0.1702 (0.0048)
Hu et al. [14]		0.0047 (0.0005)	0.9993 (0.0006)	0.4520 (0.4915)
STB	Hub	0.3067 (0.0425)	0.9986 (0.0009)	0.9218 (0.0557)
STC		0.1183 (0.0310)	0.9991 (0.0017)	0.9198 (0.1473)
ZIBR		0.1439 (0.0910)	0.9999 (0.0012)	0.4827 (0.2760)
ANCOM-BC		0.7927 (0.0154)	0.5204 (0.0168)	0.1465 (0.0032)
Hu et al. [14]		0.0025 (0.0046)	0.9991 (0.0005)	0.2194 (0.4068)
STB	Cluster	0.3255 (0.0513)	0.9974 (0.0018)	0.9065 (0.0533)
STC		0.1339 (0.0425)	0.9998 (0.0004)	0.9851 (0.0354)
ZIBR		0.1160 (0.0811)	0.9993 (0.0033)	0.3760 (0.2261)
ANCOM-BC		0.8107 (0.0155)	0.5789 (0.0228)	0.1749 (0.0073)
Hu et al. [14]		0.0034 (0.0048)	0.9992 (0.0005)	0.3280 (0.4667)

Different graph types are set to reflect various correlation structures among OTUs. Random graph structure indicates that OTUs are correlated with each other randomly. The hub and cluster graphs capture some aspects of biological networks, such as highly connected nodes and community structure. More details can be found in Osborne et al. [20]

### Comparison of STB and STC under various sparsity levels

**Fig. 2:**
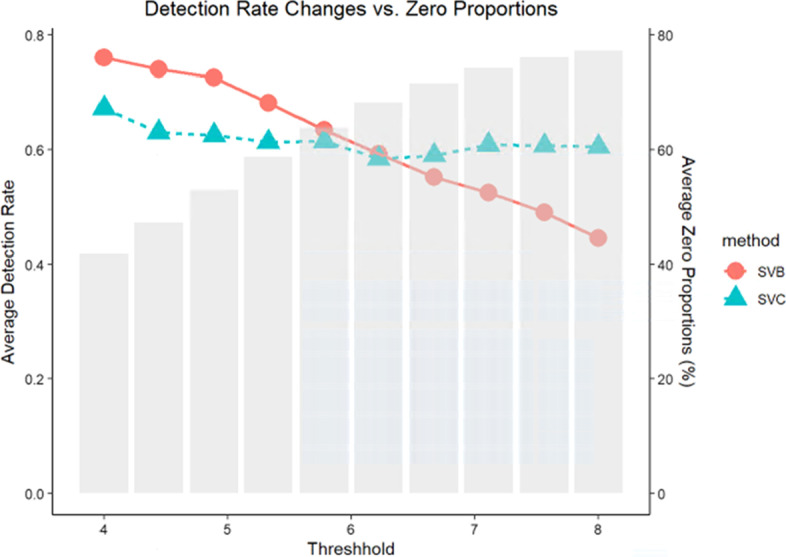
Average block-level identification rate of first four blocks between STB and STC

To provide researchers with more guidance on the utilization of STB and STC in real applications, we focus on comparing the performance of STB and STC under different sparsity levels in this section. The block-level identification rate of STB and STC under various sparsity levels are shown in Fig. 2. With the sparsity increases from 0.4 to 0.8, the average identification rates of first four blocks for both STB and STC decrease since less information are provided in the data. STB outperforms STC at lower sparsity levels (< 0.6) while STC works better in presence of higher zero proportion (> 0.6). When there are more zeros in taxa count data, the abundance of the counts will play a vital role in determining the associations, which might be the reason why STC outperforms STB in the high sparsity scenarios.

We also compare STB and STC with other methods, including ZIBR, ANCOM-BC and the original super-variant method in Hu et al. [14] as displayed in Additional file 1: Fig. S1. When the sparsity increases from 0.4 to 0.8, the average identification rates of first four blocks for ZIBR decrease but the original super-variant method is more robust to sparsity changes. Similar to the power results in the previous section, STB and STC perform better than ZIBR and the original super-variant method. While the identification rates and sensitivities are higher for ANCOM-BC, the low specificities and precisions indicate that it may predict everything into positives which limits its usefulness.

The x-axis shows the values of $a$ in the simulation studies controlling sparsity levels. With $a$ ranging from 4 to 8, the sparsity level varies from 40 to 80%. Bar plot displays the sparsity/zero proportions of simulated OTUs. Dot and line plots demonstrate the average identification rate of first four blocks versus different sparsity levels (red and circle: STB; blue and triangle: STC).

## Real data analysis

In this section, we apply our proposed method to two datasets about colorectal cancer, from Baxter et al. [4] and Zeller et al. [10], respectively. Duvallet et al. [5] preprocessed the raw 16S sequencing data into raw OTU table that are ready to use (MicrobiomeHD database: https://doi.org/10.5281/zenodo.569601). Following the filtering recommendations applied by Duvallet et al. [5], we remove samples with fewer than 100 reads and OTUs with fewer than 10 reads, as well as OTUs which are present in fewer than 1% of samples within the dataset. We calculate the relative abundance of each OTU through dividing its value by the total reads per sample. For Baxter’s data, we end up with 490 samples (CRC: 120, Health: 370) and 27, 354 OTUs, which are partitioned into 163 blocks (genus) by RDP classifier [5]. For Zeller’s data, we include 116 samples (CRC: 41, H: 75) and 82, 665 OTUs. The Baxter’s data are used as the discovery set since the dataset contains more samples, and Zeller’s data are used for verification. Compared to former work from Baxter et al. [4] which used leave-one-out and tenfold cross-validations for assessing model performance, we validate our findings by an external dataset to give rise to more reliable and convincing results. A super-taxon is chosen to be a genus consisting of related species, which can reflect evolutionally hierarchical structure. We choose genera-level analyses since it is the highest level that we can get for taxonomy assignments. Additionally, most previous studies [4, 5, 10] also focused on genera-level analyses, which can provide us a more reasonable comparison. However, depending on the interest of researchers, other hierarchical levels can be used. We end up with 163 genera, leading to the significance level for discovery set as 0.05/163 = 3.07e-4.

Since we need to randomly divide the discovery set into two even part to control type-I error as we claim in the simulation studies, to ascertain the stability of the associations, we repeat the division 10 times and retain the significant super-taxa and their contributing OTUs at each repetition. Typically, microbiota-disease association analyses do not include an internal assessment, but we replicate our procedure 10 times as a safeguard strategy for detecting potential and stable signals without dramatically increasing the computational burden. Finally, for super-taxa that are consistently selected across multiple repeats (at least 2 out of 10 repetitions), we conduct analyses in the complete discovery dataset (Baxter’s dataset) and verification set (Zeller’s dataset) to further validate their associations.

For verification, since the OTU sequence lengths in two datasets are different (Baxter: 250 bp, Zeller: 200 bp), we need to find the OTU sequences in the verification dataset that align with significant OTU sequences in the discovery set. To achieve this, we apply BLAST algorithm [23–25] to calculate the sequence identity score between two datasets. The identity score is proved to be a valid measure of sequence similarity [26]. Specifically, for each significant OTU in the discovery set, we find the OTU in the verification set that has the largest sequence identity score with that significant OTU. The significance level is 0.05 divide by number of significant blocks in the discovery set.

For the results of 10 repetitions, under STB framework, both block 84 and block 107 are selected in all repetitions. STC performs similarly to STB, where it identifies block 84 in all repetitions and block 107 in 9 out of 10 repetitions. We validate the blocks that are frequently selected to representatively form 2 super-taxa, including block 107 and block 84 for both STB and STC. Tables 4 and 5 give their estimated effects on complete discovery set and verification set for STB and STC respectively. Only block 107 is significant in the verification set (p = 1.55e-04) under STB framework while the two blocks are both validated to be significant in the Zeller’s dataset (block 107: p = 8.21e-06; block 84: p = 0.0084) by using STC.

**Table 4:** Real data results for STB

(a)				
Super-taxon	Discovery		Verification	
	Odds ratio (95% CI)	P-value	Odds ratio (95% CI)	P-value
Block 107	8.4851 (4.3838, 16.4234)	2.21e-10	4.2538 (2.0093, 9.0057)	1.55e-04
Block 84	5.5873 (3.2172, 9.7034)	1e-09	1.7073 (0.9901, 2.9441)	0.0543

(b)			
OTU	Super-taxon	Genus	Species
denovo670	Block 107	Peptostreptococcus	Peptostreptococcus anaerobius
denovo1172	Block 107	Peptostreptococcus	Peptostreptococcus stomatis

Marginal Effects of 2 Super-taxa on Baxter’s dataset (discovery set) and Zeller’s dataset (verification set) are displayed in Table 4. The selected OTUs and their mapping to species are in Table 4

**Table 5:** Real data results for STC

(a)				
Super-taxon	Discovery		Verification	
	Odds Ratio (95% CI)	P-value	Odds Ratio (95% CI)	P-value
Block 107	9.2060 (4.4911, 18.8705)	1.35e-09	9.0996 (3.4478, 24.0160)	8.21e-06
Block 84	5.5723 (3.1147, 9.9690)	7.11e-09	3.4140 (1.3696, 8.5101)	8.42e-03

(b)			
OTU	Super-taxon	Genus	Species
denovo670	Block 107	Peptostreptococcus	Peptostreptococcus anaerobius
denovo1172	Block 107	Peptostreptococcus	Peptostreptococcus stomatis
denovo596	Block 84	Parvimonas	Parvimonas micra
denovo3935	Block 84	Parvimonas	Parvimonas sp.
denovo8735	Block 84	Parvimonas	Parvimonas sp.

Marginal Effects of 2 Super-taxa on Baxter’s dataset (discovery set) and Zeller’s dataset (verification set) are displayed in Table 5. The selected OTUs and their mapping to species are in Table 5

For OTU-level identification, STB identifies two OTUs, denovo670 and denovo1172 for block 107. For STC, it discovers the same sets OTUs for block 107. Additionally, it also uncovers three OTUs for block 84, including denovo596, denovo3935 and denovo8735. For better biological interpretation, the identified OTUs are assigned to a specie according to NCBI BLAST [27], and the assignments are shown in the second table in Tables 4 and 5.

To further validate the joint effects of multiple OTUs within a block, we compare the Akaike information criterion [28] for regressions only including individual OTU with the one with our super-taxon. For STC, the AIC values are 498.0397 and 518.7474 for block 107 and block 84, respectively. Meanwhile, the minimum AIC for regression models with individual OTU is 529.2436 based on denovo596, which implies that the model based on super-taxon (joint effects) identified by our approach better fits the data. By further comparing the odds ratios (OR), we observe that OR is much higher for the model with super-taxon, indicating that the presence and enrichment of multiple OTUs may result in higher risk of CRC. Combining results from both methods, we finally identify two genera: Parvimonas and Peptostreptococcus with five OTUs belonging to four species: Peptostreptococcus stomatis, Peptostreptococcus anaerobius, Parvimonas micra and Parvimonas sp.which are significantly associated with CRC.

## Discussion

In this study, we propose a new concept called super-taxon to group several OTUs together based on hierarchical information of taxa as the joint effect factor. Associations between the super-taxon and the disease are expected to be more stable than that of a single OTU and may enhance the power of detection. We propose the average of non-zero and binary transformation of OTUs involved in a super-taxon as translated value to reduce the effect of un-presented taxa. In simulations, we demonstrate that the proposed method can be more powerful than the other association method in the dataset with lots of zeros and signals with correlated structure. We also provide empirical suggestions that STC outperforms STB when lots of zeros are presented in the data. Finally, we apply our method to two datasets for colorectal cancer, one as discovery set and the other as verification set to generate reliable results. We repeat the analyses for 10 times to consolidate more convincing and trustworthy findings and results. Our methods uncover several colorectal cancer associated genera from a fresh angle as well as identify the novel joint effects of two groups of OTUs for further investigation.

In the simulation studies, we notice that STB outperforms STC when sparsity in the data is low while STC works better in presence of high sparsity. In practice, we suggest that researchers should try both STB and STC, but more attention should be focused on trying STB for less sparse data and STC for data with more zeros since STC can take advantage of the abundance information of OTUs in such case.

For real data application, the average sparsity of the two datasets is around 90%. Based on our simulation results, STC may perform better than STB, which agrees with what we have observed. Besides, there are overlapping between the findings from STB and STC, which further validates our approaches.

We identify the associations of Peptostreptococcus genus and Parvimonas genus with CRC [29, 30], which have been reported in previous studies [5, 31]). In the paper of Duvallet et al. [5], they grouped OTUs by taking the summation of the abundance across those OTUs and performed univariate test from group-level. However, such methods are unable to detect individual OTU and their joint effects on diseases. Though Shal et al. [31] performed random forest analyses at OTU-level, which could potentially capture the joint effects, they were unable to provide an estimation of joint effect sizes that can be achieved through our approach. On top of the two genera, we also report the joint effect of Peptostreptococcus stomatis and Peptostreptococcus anaerobius, as well as joint effect of Parvimonas micra and Parvimonas sp. on CRC. We demonstrate that by considering joint effects, the model fits datasets better with lower AIC values. We also reveal that the presence and enrichment of multiple OTUs might result in higher risk of CRC in marginal logistic regressions.

The composition of OTUs in each taxonomy levels (Class, Order, Family and Genus) are added to provide more insights on different hierarchical levels and associated effects on CRC. For both class and order level, there is only one class (Clostridia) and one order (Clostridiales) that are selected over twice across 10 iterations with the STC method in the discovery set. But they are not verified on the external validation set (p = 0.4220). In terms of family level, STC identifies the Clostridiales_Incertae Sedis XI family 8 times out of 10 iterations which is further verified on the validation set (OR = 3.58, p = 0.0081). The Clostridiales_Incertae Sedis XI family consists of six OTUs (Additional file 1: Table S1). Apart from Parvimonas reported at genera-level, there are two more genera identified, Anaerococcus and Peptoniphilus, indicating potential interactive effects among three genera on CRC. Our recommendation is that there is no best hierarchy to use in real practice and balance exists between higher taxonomic resolution and lower detection power, which can depend on different needs and scientific relevance of microbiome studies. On the one hand, coarsen scale of taxonomy may lead to a model losing interpretability power. On the other hand, focusing on high resolution data may fail to detect associations between aggregated taxonomic units and disease manifestation should they exist at low resolution levels. Based on our findings, family or genera-level can give more meaningful and interpretable results. In summary, our approach allows researcher to use hierarchical information to learn how microbes function as a community for disease manifestation and provides estimated joint effect size for increasing disease risks.

The marginal biological effects of Peptostreptococcus stomatis, Peptostreptococcus anaerobius, Parvimonas micra and Parvimonas sp. on CRC have been studied previously. Parvimonas micra can disrupt the normal functioning of the NOD2 signaling pathway in periodontitis [32], which could potentially lead to a protumorigenic and inflammatory environment. One previous study indicates that Parvimonas sp. Oral taxon 110 and Parvimonas sp. Oral taxon 393 are enriched in CRC patients’s microbiota [33]. Studies have shown that patients with Peptostreptococcus stomatis (P. stomatis) and Peptostreptococcus anaerobius (P. anaerobius) have an increased risk of developing CRC [34, 35]. P. stomatis is a mild saccharolytic and fermented product producer, including acetic, isobutyric, isovaleric, and isocaproic acids [36]. It might contribute to the acidic and hypoxic tumor microenvironment, which supports bacterial colonization. P. anaerobius enrichment in CRC patient stool and tissue has been validated by a previous study [35]. It also reports that P. anaerobius interacted with Toll-like receptor 2 (TLR2) and Toll-like receptor 4 (TLR4) on colon cells to reactive oxygen species accumulation, which supports cholesterol synthesis and cellular proliferation.

In addition, we find that two identified OTUs, denovo3935 and denovo8735 for Parvimonas genus belong to the same species, Parvimonas sp., which might imply that the potential sub-species exist.

For the additional two genera identified at family level, Anaerococcus and Peptoniphilus, Anaerococcus is more prevalent in patients with CRC compared to controls [37]. Peptoniphilus is also observed to be enriched in CRC patients [38]. Anaerococcus, Parvimonas, Peptoniphilus and Peptostreptococcus are all gram-positive anaerobic cocci (GPACs) [39]. One of the species in Anaerococcus genus, Anaerococcus prevotii can produce urease in the gastrointestinal tract (GI) that interrupts the nitrogen recycling and results in products including ammonia, which are harmful to the host health. One species Peptoniphilus asaccharolyticus under Peptoniphilus genus has similar same metabolism in the host GI [40]. The analogous mechanism of two genera is an indication of their joint effects on CRC development and motivates us for future investigation.

Although there are some arguments on the high noise and low quality of the sequence data especially for species-level or beyond, with the advanced sequencing technology, our methods will provide a valid and useful tool to go beyond species-level to identify strains of bacteria, and even genomic variants in those strains [41] as well as their associations with diseases. Apart from that, the compositionality feature of OTUs poses challenges for analysis using standard conventional statistical procedures, including non-independency of OTUs and potential interactions between them. In this sense, mass univariate regression commonly used in genome-wide association studies is not feasible. One may adopt regression-based method and account for the unit sum of the covariates simultaneously [42]. However, this approach requires imposing a linear constraint on regression coefficients, which complicates computation and may not take interaction terms into account. Nevertheless, we consider tree-based method as a sensible choice in our study. With the application of random forest model, it can deal with autocorrelations between OTUs and alleviate compositionality problems as suggested by Ranganathan and Borges [43] and Knights et al. [44]. Other works using random forest to develop predictive model for microbiome data include Baxter et al. [4] and Duvallet et al. [5] for studying CRC.

The most important finding of our work is the identification of the joint effects between P. anaerobius and P. stomatis, and that between Parvimonas micra and Parvimonas sp. Although the marginal effects of individual species such as P. stomatis and P. anaerobius on CRC have been reported and potentially joint effects between them were studies by Shah et al. [31], to the best of our knowledge, there have been few attempts on unrevealing their joint effects. Hence, identifying joint effects of OTUs and providing an estimation of joint effect sizes on CRC are of great importance to potentially decrease cancer risk and perhaps even improve diagnosis, treatment stratification, and therapy. One possible explanation of the joint effect between P. anaerobius and P. stomatis is that P. stomatis leads to the intestinal dysbiosis of P. anaerobius in CRC patients. P. stomatis is capable of producing lysylphosphatidylglycerol (LPG), a major component of the bacterial membrane. LPG synthesis contributes to bacterial virulence since it participants in the resistance mechanism against cationic antimicrobial peptides produced by the host’s immune system and by competing microorganisms [45]. The competition could contribute to the intestinal dysbiosis of P. anaerobius by either mitigating inflammation through reducing indole-3-propionic acid (IPA) and indoleacrylic acid (IA) levels [46] or affecting other symbiotic microbes such as Prevotella bivia [47]. However, no functional study on joint effects between P. stomatis and P. anaerobius or between Parvimonas micra and Parvimonas sp. in colorectal tumor development exist to date, which requires further research and attention.

As a final remark, we should note that although the proposed method enjoys important advantages, it can be extended and improved. For instance, it may be helpful to incorporate more hierarchical structure information to better group the OTUs. In addition, the sample size of real datasets adopted here is limited to draw more significant conclusions.

## Conclusions

In this paper, we propose a new concept called super-taxon for microbiota-disease association studies, and present STB and STC to identify joint effects of microbiomes on CRC. Compared to state-of-the-art differential abundance analysis approaches, STB and STC yield better identification performance in situations where microbes are highly correlated. We discover and verify two known genera with CRC while innovatively identify and estimate joint effects of multiple OTUs on CRC. These findings consolidate benefits of proposed approaches and provide potential directions for better understanding the roles of microbes in CRC.

## Supplementary Information

**Additional file 1**. Simulation Results under various sparsitiy levels and CRC associated microbes from family level.

## Data Availability

The datasets generated and analyzed during the current study including the raw processed OTU tables are available at the MicrobiomeHD database: https://doi.org/10.5281/zenodo.569601 [5]. The code our method in a practical analytical pipeline for has been made publicly available at https://github.com/daiw3/STB-STC. All other relevant data supporting the findings of the study are available in this article or from the corresponding author on request.
