# Supplementary material for: Super-taxon in human microbiome are identified to be associated with colorectal cancer
Source: BMC Bioinformatics. 2022 Jun 21;23:243. doi: 10.1186/s12859-022-04786-9 (PMC9215102; doi:10.1186/s12859-022-04786-9)
Supplement: Supplementary file 1 — Additional file 1. Simulation Results under various sparsitiy levels and CRC associated microbes from family level. [file 12859_2022_4786_MOESM1_ESM.docx]

**Additional file 1s**

**Figure S1** Results of average block-level detection rate, sensitivity, specific and precision varying by sparsity levels. The x-axis shows the values of $a$ in the simulation studies controlling sparsity levels. With $a$ ranging from 4 to 8, the sparsity level varies from 40% to 80%. The bar plot and right y-axis display the sparsity / zero proportions of simulated OTUs. Different approaches are denoted by colors and shapes. (a): the average identification rate of first four blocks versus different sparsity. (b): the average sensitivity in identifying true blocks under various sparsity levels. (c): the average specificity in identifying true blocks under various sparsity levels. (d): the average precision in identifying true blocks under various sparsity levels


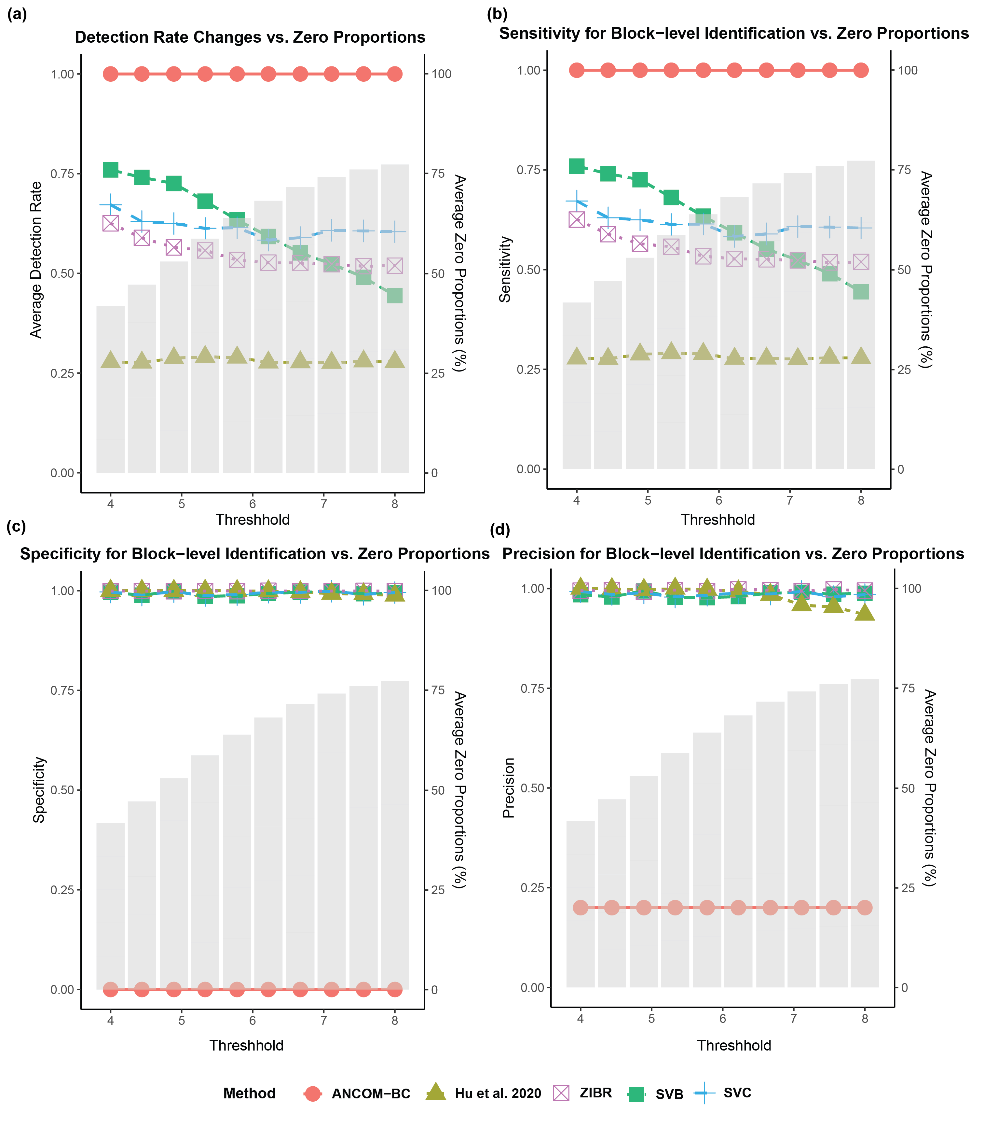


**Table S1** The selected OTUs and their mapping to families and genus

| OTU | Super-taxon | Family | Genus |
| --- | --- | --- | --- |
| denovo596 | Block 28 | Clostridiales_Incertae Sedis XI | Parvimonas |
| denovo3935 | Block 28 | Clostridiales_Incertae Sedis XI | Parvimonas |
| denovo947 | Block 28 | Clostridiales_Incertae Sedis XI | Anaerococcus |
| denovo5938 | Block 28 | Clostridiales_Incertae Sedis XI | Anaerococcus |
| denovo1208 | Block 28 | Clostridiales_Incertae Sedis XI | Peptoniphilus |
| denovo10088 | Block 28 | Clostridiales_Incertae Sedis XI | Peptoniphilus |
